# P-1861. Increased Disease Severity in Experimental *Clostridioides difficile* Infection is Associated with Increased Levels of Fecal Toxin

**DOI:** 10.1093/ofid/ofae631.2022

**Published:** 2025-01-29

**Authors:** Hannah Ruppel, Michael Dieterle, Kimberly Vendrov, Alexandra Standke, Vincent B Young, Krishna Rao

**Affiliations:** University of Michigan, Ann Arbor, Michigan; University of Michigan, Ann Arbor, Michigan; University of Michigan, Ann Arbor, Michigan; University of Michigan, Department of Internal Medicine, Division of Infectious Diseases, Ann Arbor, MI; University of Michigan, Ann Arbor, Michigan; Department of Internal Medicine, Infectious Diseases Division University of Michigan, Ann Arbor, Michigan, Ann Arbor, MI

## Abstract

**Background:**

*Clostridioides difficile* is a toxin-producing bacteria that is the most common cause of nosocomial diarrhea. The CDC estimates that there are around 500,000 infections caused by *C. difficile* each year and has declared that the bacteria is an urgent public health threat . The production of toxin is thought to be the primary driver of disease pathogenesis. To understand the relationship between toxin and disease severity, we analyzed the correlation between clinical and histopathologic scores and toxin amount using a murine model.

**Figure:**
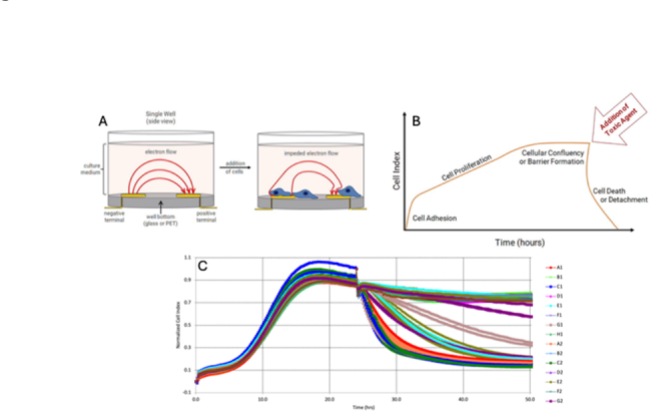

Real Time Cellular Analysis utilizing Aligent Systems. (A) Single well view of well before (left) and after (right) addition of HT-29 cells. Cellular impedance is measured using electron flow from the negative terminal to the positive terminal. Increased cell proliferation and adhesion to the bottom of the well leads to increased cellular impedance. (B) Example graphic of real-time cellular index tracing. (C) Experimental real-time cellular index tracing of fecal samples and toxin standards.

**Methods:**

Young (6- to 8-week-old), specific-pathogen-free mice were treated with 10 days of Cefoperazone to render them susceptible to *C. difficile* infection. Mice were given an oral gavage of either water, C. *difficile* VPI10463 spores, or *C. difficile* 630g spores. We monitored mice throughout the experiment for posture, coat, diarrheal signs, activity, and weight change to determine clinical score. At the time of sacrifice, tissue was collected and histopathologic scores were determined based on edema, epithelial damage, and inflammatory cell infiltration. Cytokines were measured using a customized Luminex panel. Real time cellular analysis was utilized to quantify the amount of toxin present in mouse fecal samples. Statistical analysis was completed in R.

**Figure:**
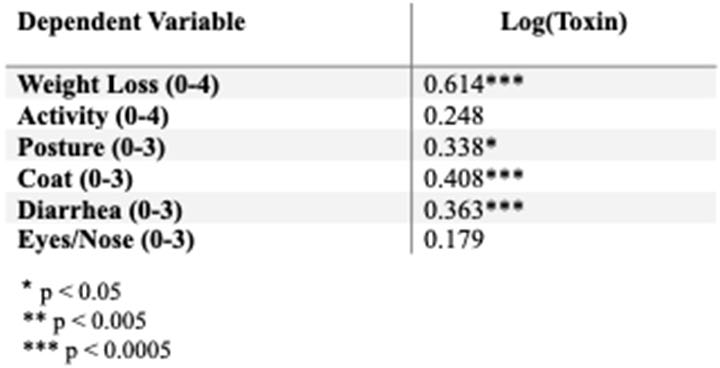

Pearson’s product-moment correlations for the relation of log(toxin) amount and individual components of clinical score

**Results:**

There was a strong, positive correlation between clinical score and toxin. When further analyzed, we found that the weight loss component of clinical score was most correlated with toxin amount (Table 1). Additionally, there was a strong, positive correlation between total histopathologic score and toxin (Figure 2). We found that all components of total histopathologic score were strongly correlated with toxin. We found multiple cytokines were correlated with toxin (Table 2).

**Figure:**
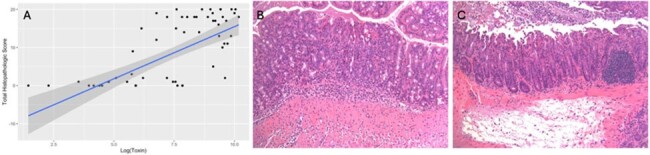

Histopathology (A) Scatterplot showing total histopathologic score (based on edema, epithelial damage, and inflammatory cell infiltration of the cecum and colon) and log(toxin) amount. Total histopathologic score has a strong, positive, correlation with log(toxin) amount (r=0.653). (B) Histopathology representative of mice without C. difficile infection. (C) Histopathology representative of mice with C. difficile infection.

**Conclusion:**

Increased toxin present in fecal samples was found to be correlated with severity of disease. Previous work established a predictive model of CDI utilizing cytokines. We expect that the relationship between toxin and disease severity is mediated by the host immune response. Understanding the relationship between toxin and disease severity could be helpful in predicting CDI prognosis and helpful in guiding therapy.

**Figure:**
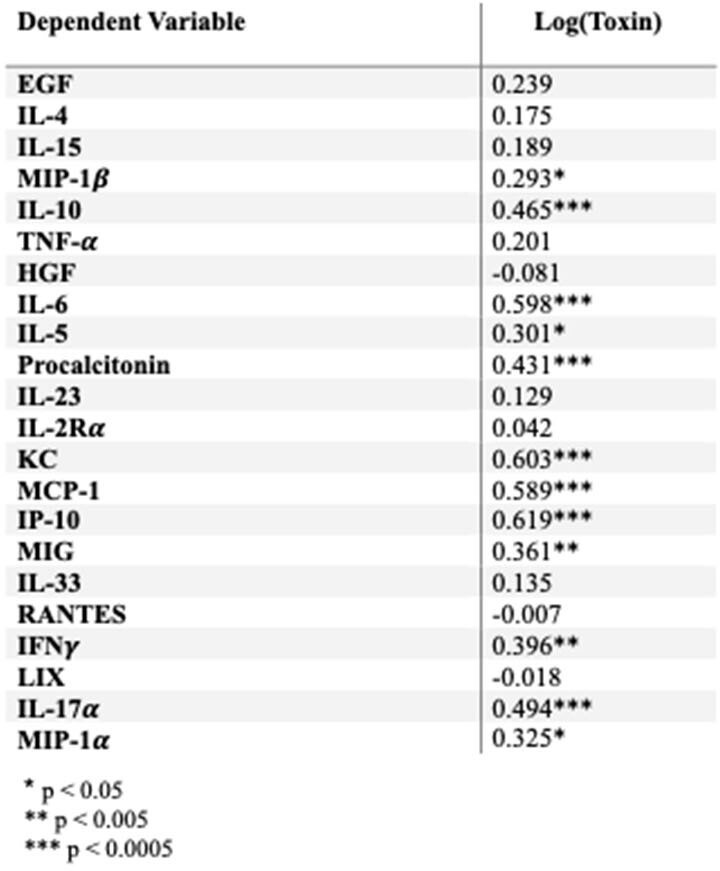

Pearson’s product-moment correlations for the relation of log(toxin) amount and individual cytokines.

**Disclosures:**

Vincent B. Young, MD, PhD, Aimmune: Honoraria|American Society for Microbiology: Board Member|Debiopharm: Advisor/Consultant|Peggy Lillis Foundation: Board Member|University of Oklahoma COBRE: Advisor/Consultant|Vedanta: Advisor/Consultant|Vedanta Bioscience: Advisor/Consultant|Vedanta Bioscience: Grant/Research Support Krishna Rao, MD, MS, Vedanta: Advisor/Consultant

